# The effect of coarctation degrees on wall shear stress indices

**DOI:** 10.1038/s41598-021-92104-3

**Published:** 2021-06-17

**Authors:** Deniz Rafiei, Mohammad Amin Abazari, M. Soltani, Mona Alimohammadi

**Affiliations:** 1 Department of Mechanical Engineering, K. N. Toosi Univeristy of Technology, Tehran, Iran; 2 Department of Electrical and Computer Engineering, Faculty of Engineering, School of Optometry and Vision Science, Faculty of Science, University of Waterloo, Waterloo, Canada; 3 Advanced Bioengineering Initiative Center, Multidisciplinary International Complex, K. N. Toosi University of Technology, Tehran, Iran; 4 Centre for Biotechnology and Bioengineering (CBB), University of Waterloo, Waterloo, ON Canada; 5 Cancer Biology Research Center, Cancer Institute of Iran, Tehran University of Medical Sciences, Tehran, Iran

**Keywords:** Aortic diseases, Mechanical engineering, Biomedical engineering

## Abstract

Coarctation of the aorta (CoA) is a congenital tightening of the proximal descending aorta. Flow quantification can be immensely valuable for an early and accurate diagnosis. However, there is a lack of appropriate diagnostic approaches for a variety of cardiovascular diseases, such as CoA. An accurate understanding of the disease depends on measurements of the global haemodynamics (criteria for heart function) and also the local haemodynamics (detailed data on the dynamics of blood flow). Playing a significant role in clinical processes, wall shear stress (WSS) cannot be measured clinically; thus, computation tools are needed to give an insight into this crucial haemodynamic parameter. In the present study, in order to enable the progress of non-invasive approaches that quantify global and local haemodynamics for different CoA severities, innovative computational blueprint simulations that include fluid–solid interaction models are developed. Since there is no clear approach for managing the CoA regarding its severity, this study proposes the use of WSS indices and pressure gradient to better establish a framework for treatment procedures in CoA patients with different severities. This provides a platform for improving CoA therapy on a patient-specific level, in which physicians can perform treatment methods based on WSS indices on top of using a mere experience. Results show how severe CoA affects the aorta in comparison to the milder cases, which can give the medical community valuable information before and after any intervention.

## Introduction

Aortic coarctation (CoA) is a congenital heart disease (CHD). Being the fifth most common CHD^1^, CoA makes up about 5–8% of all congenital cardiac deficiencies^2, 3^. It is identified by narrowing the upper descending aorta, mostly after the beginning of the left subclavian artery, affecting the blood flow from the heart to all body. As nearly 60% of the aorta's buffer capacity is located in the proximal aorta^4^, this local strengthening controls the capacitance of the aorta. Moreover, stiffening and a local narrowing causes wave reflections quickly reaching the heart^5^. Afterward, it is not difficult to imagine that blood pressure distribution and perfusion changes due to the obstruction; leading to cerebral and upper body hypertension, left ventricular hypertrophy, coronary artery disease, cerebral hemorrhage, stroke, and aortic ruptures, aneurysm formation, and decreased life expectancy^6–9^. Regrettably, all available treatment strategies (i.e. surgical correction or endovascular procedures) might contribute to potential early and late complications which again influence on the future morbidity and mortality of CoA population^10–12^. Depending on the severity of the obstruction and other comorbidities, the early course of disease might be asymptomatic, but with the CoA severity progression, heart failure increases among 60% of adults over 40 with untreated CoA. 75% of these patients die by 50, and 90% die by 60^13^. Indeed, despite advances in imaging and interventional techniques, the knowledge regarding the long-term benefits of current therapeutic approaches and the advantages of each scenario are obscure. While all of these can provide useful information on the cardiac deterioration and heart regeneration of the patients, clinical decisions are currently based on medical imaging^14^, that those techniques do not address this accurately^15^. “Cardiology is flow,”^16^ hence, the major causes of CoA morbidity could be described based on adverse haemodynamics. With the advance in computing power, computational simulations can provide meaningful insights into the biomechanics of CoA, presenting data behind the pressure failure observed during the Doppler Ultrasound examination, which is challenging to attain in-vivo. Many decades ago, O’Rourke and Cartmill proposed that the majority of unhealthiness for CoA could be described on the base of unusual haemodynamics through the ascending aorta and connected branches by indicating its alarmingly altered conduit (blood flow) and cushioning (capacity) functions linking to CoA^17^. A thorough search of the relevant literature yielded that there is no clear interventional guideline for clinicians when treating CoA. Currently, the length of the selected stent should cover the entire lesion, extending from the beginning of the left subclavian artery or the left common carotid artery to approximately 15 mm beyond the CoA region^18, 19^.

The decision on the optimal operation procedure is complicated, and there is no evidence-based standard of care. Studies show that the rate of recoarctation varies from 3 to 15%^20, 21^. Patients suffer from the formation of aneurysm or dissection, stent fracture, or the re-obstruction within the stent^22^. Also, the development of aneurysms following intravascular stent placement has been previously described in many studies^15, 23–26^. What is more, paraplegia can occur by interventional repairing after operations for coarctation^27^.

Therefore, this study proposes the use of WSS indices and pressure gradient to better establish a framework for stent placement in CoA patients with different severities, which could lead to less stent failures and follow-up complications. Being a vector quantity, WSS possesses both magnitude and direction-two components, affecting the advancement and progression of the aortic arch malady^28^. Effective management of CoA relies on not only the quantifications of the global haemodynamics (heart workload and instantaneous pressure) but also of the local haemodynamics (detailed data of the flow dynamics). Despite existing great studies on computational modeling of the coarctation of the aorta, most studies do not cover the aorta’ elasticity and the fluid–structure interaction (FSI)^29–36^ and/or have focused on the haemodynamic effect of CoA in patient-specific cases^37, 38^ or on arterial WSS^39, 40^. Studies showed that the inclusion of the vessel wall has a great impact on the blood flow parameters and WSS indices. In fact, the rigid wall design overestimates the time-averaged wall shear stress (TAWSS) by more than 50%^41^. The complexity of aortic flow patterns, choosing relevant and realistic boundary conditions, imitating vascular compliance, and the vessel’s motion all contribute to the rarity of such studies. The present study evaluates the proportion of flow post-coarctation to better understand the affected and altered haemodynamic parameters caused by the area reduction in the throat of CoA that would help clinicians introduce the intervention framework based on WSS indices and pressure gradient, which has not been investigated before. In conclusion, this study emphasizes; the importance of choosing the optimum treatment methods for CoA patients considering its severity (as considerable differences of disturbed hemodynamics are found for different CoA severities in this study), and the great impact of doing FSI simulations for each patient and considering the altered WSS indices (as several stent failure and complications have been reported). This approach is based on logical engineering equations rather than utilizing stents based on experience. Results show how severe CoA affects the aorta in comparison to the milder cases, which can give valuable insights for clinical usage and predictions and repair planning. Additionally, there is no idealized FSI CoA geometries study that investigates the disturbed hemodynamics, which can give a reasonable view of this study’s aim without the impact of the different morphological factors on important hemodynamics parameters.

## Methods

### Aortic geometry

**Figure 1:**
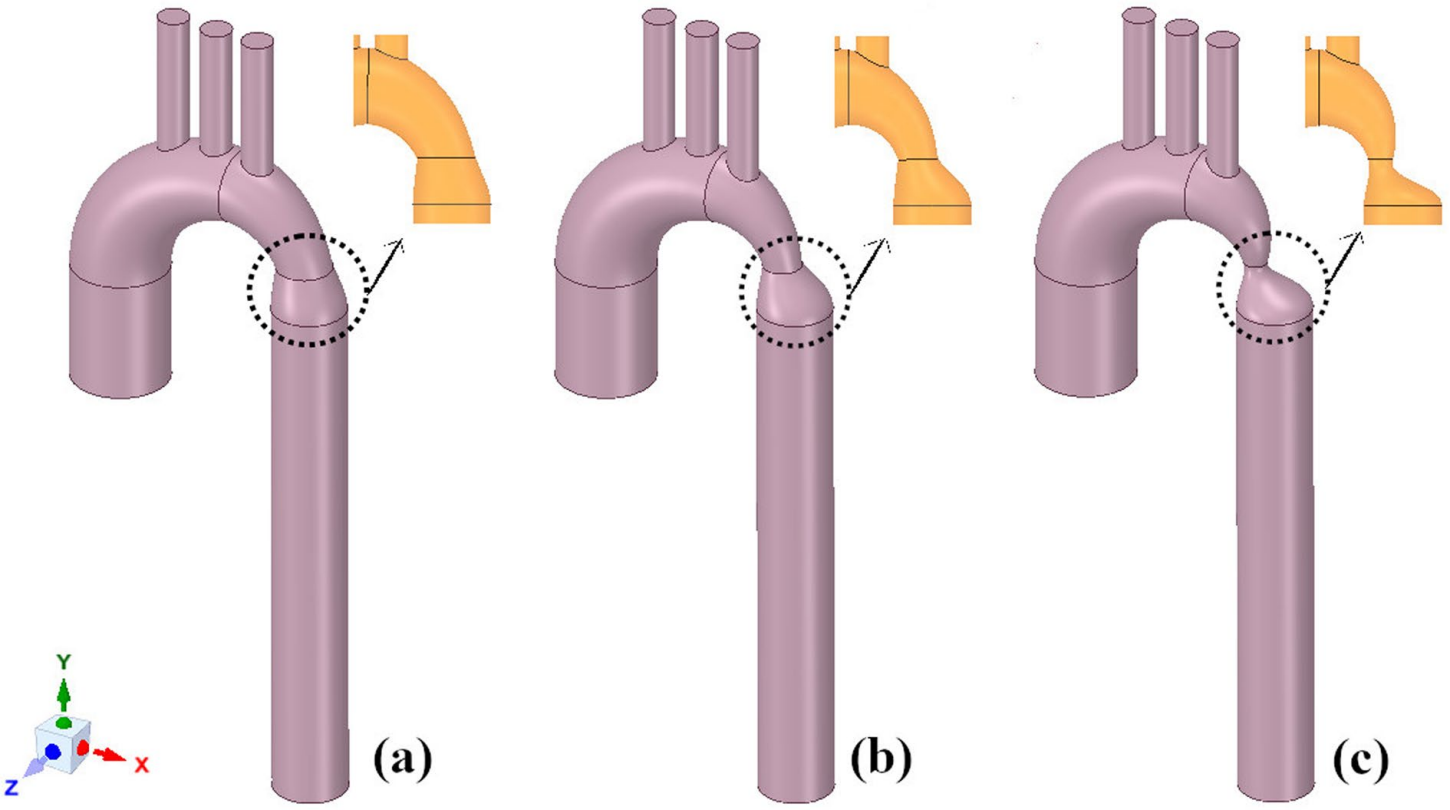
Three idealised geometries. (**a**) 25% coarctation; (**b**) 50% coarctation; and (**c**) 75% coarctation.

Three coarctation degrees of mild, moderate, and severe have been considered, and their haemodynamic parameters have been compared. The main concept of the geometry is taken from the study (Larissa Hütter et al.)^42^, with the 27.5 mm diameter of the ascending aorta, 156 mm length of the descending aorta, and the wall thickness of 1.6 mm. The relevant tapering of each geometry has been applied based on the cross-sectional area with 25%, 50%, and 75% narrowing demonstrated in Fig. 1. The degree of coarctation (CD) was defined as a ratio of cross-sectional areas: CD = $100\% \times \left( {1 - A_{{CoA}} {\text{/}}A_{{Desc}} } \right),$ where $A_{{CoA}}$ and $A_{{Desc}}$ are the cross-sectional areas at the coarctation and at the descending aorta, respectively. Efforts have been made to ensure that three models are the same except in their severity of narrowing.

### Boundary conditions

The governing equation for the blood flow and the motion of the vessel wall is solved using ANSYS-CFX (ANSYS Inc., PA, USA), providing two-way FSI models for all three cases. The fluid domain was discretized using Ansys meshing with about 500,000 high-quality tetrahedral mesh elements. To minimize the computational errors near the wall, fifteen prism layers (1.2 growth rate) with dimensionless height of the near wall cells ($y^{ + }$) < 1 were applied. For the sensitivity analysis, two more mesh elements were calculated; however, the current model was chosen due to its accuracy and computational efficacy. For the purpose of additional accuracy and saving computational time, a mesh refinement block is deployed on the coarctation region, providing an additional refinement in the flow domain distal to the CoA. As Doormaal et al. indicated the superiority of using magnetic resonance imaging (MRI) based inlet velocity profiles over an idealised profile in the mouse aortic arch to obtain more accurate results of haemodynamic distributions^43^, the same measured time-varying flow rate profiles and pressure waves were mapped as boundary conditions to the ascending and descending aorta in addition to the three side branches in all three cases taken from a coarctation study^44^. The three-dimensional CFD models were discretised by tetrahedral elements to provide an accurate display of cross-section averaged measures, including flow and the loss of pressure for each branch. As resembling blood; the fluid is considered a Newtonian, incompressible fluid, with the density of $\rho = 1056\;{\text{kg/m}}^{3}$ and dynamic viscosity of μ = 0.0035 Pa s^45^. Blood flow in healthy vessels is regularly laminar and does not undergo any turbulence. However, in the presence of flow obstruction, such as CoA, turbulence develops in the aorta. A shear stress transport (SST) turbulence model with 1% turbulence level at the inlet was employed. And the time step size is 0.04 s. Mass Flow Rate and Pressure waves were modeled as a function of time (Mercuri, M.), and Vessel wall displacements were defined as a function of the blood pressures and velocities at the fluid–wall interface using FSI simulation. A two-way coupled method is applied, and aorta’s properties are added through the transient structural. For all calculations, flow velocity has been assumed zero at the vessel's luminal wall, satisfying a so-called no-slip boundary condition. The continuity and Navier–Stokes equations solved in 3D using a transient analysis shown in “Eqs. (1) and (2)” respectively:

1 $$\nabla \cdot \vec{u} = 0$$

2 $$\rho \left[ {\frac{{\partial \vec{u}}}{{\partial t}} + \left( {u \cdot \nabla } \right)\vec{u} + \nabla p - \mu \nabla ^{2} \vec{u}} \right] = 0$$

where $\vec{\text{u}}$, ρ, p, and μ defines the vector of fluid velocity, density, the hydrostatic pressure, and viscosity of blood, respectively. Blood flow velocity and pressure, wall displacement, and streamlines are shown at three cardiac-cycle points of mid-systole (t = 0.12 s), peak systole (t = 0.2 s), and dicrotic notch (t = 0.44 s), and after that, demonstrated and compared among the three cases. The aortic tissue's material behavior characterised using a linear elastic model obtained from the studies^46, 47^ (with the density of 1160 ${\text{kg/m}}^{3}$ and young’s modulus $1.08 \times 10^{6}$ Pa and Poisson's ratio 0.49). Although the stiffness over the aorta is not constant, and it raises with the narrowing, the average stiffness is considered for this study. Wall shear stress (WSS) is the instantaneous stress applied at the wall and in order to derive a meaningful conclusion, the average of WSS (TAWSS (see for^48^ definition)) over a cardiac cycle of 1.27 s is calculated.

3 $$TAWSS = \frac{1}{T}\int \left| {\overrightarrow {{WSS\left( t \right)}} } \right|dt$$

In order to assess the temporal oscillations in the immediate WSS vector over the cardiac cycle, the oscillatory shear index (OSI) has been used as follows: (See^49^ for definition)

4 $$OSI = 0.5\left( {1 - \frac{{\left| {\mathop \int \nolimits_{0}^{T} \overrightarrow {{WSS\left( t \right)}} dt} \right|}}{{\mathop \int \nolimits_{0}^{T} \left| {\overrightarrow {{WSS\left( t \right)}} } \right|dt}}} \right)$$

A particular index, HOLMES, is proposed in light of the signs of raised infiltration in low oscillatory areas^50^ (Highly Oscillatory and Low Magnitude Shear), which is known as a useful index for linking these two features^51^.

5 $${\text{HOLMES}} = {\text{TAWSS}}\left( {0.5 - {\text{OSI}}} \right)$$

The HOLMES indicator is the modified version of TAWSS. The (0.5 − OSI) term cuts back the index in areas of low, oscillatory WSS. Besides, HOLMES offers a linear proportional index to TAWSS, which instinctively corresponds to shear features' detected effects on endothelial permeability.

The relative residence time (RRT) is calculated as^52^:

6 $${\text{RRT}} = \frac{1}{{\left( {1 - 2 \cdot {\text{OSI}}} \right) \cdot {\text{TAWSS}}}}$$

RRT calculates the duration of residence of particles close to the wall, and it is a single measure of oscillating and low shear stress. Being inversely proportional to the magnitude of the TAWSS vector, RRT has apparent links to atherosclerosis.

## Results

### Wall displacement

**Figure 2:**
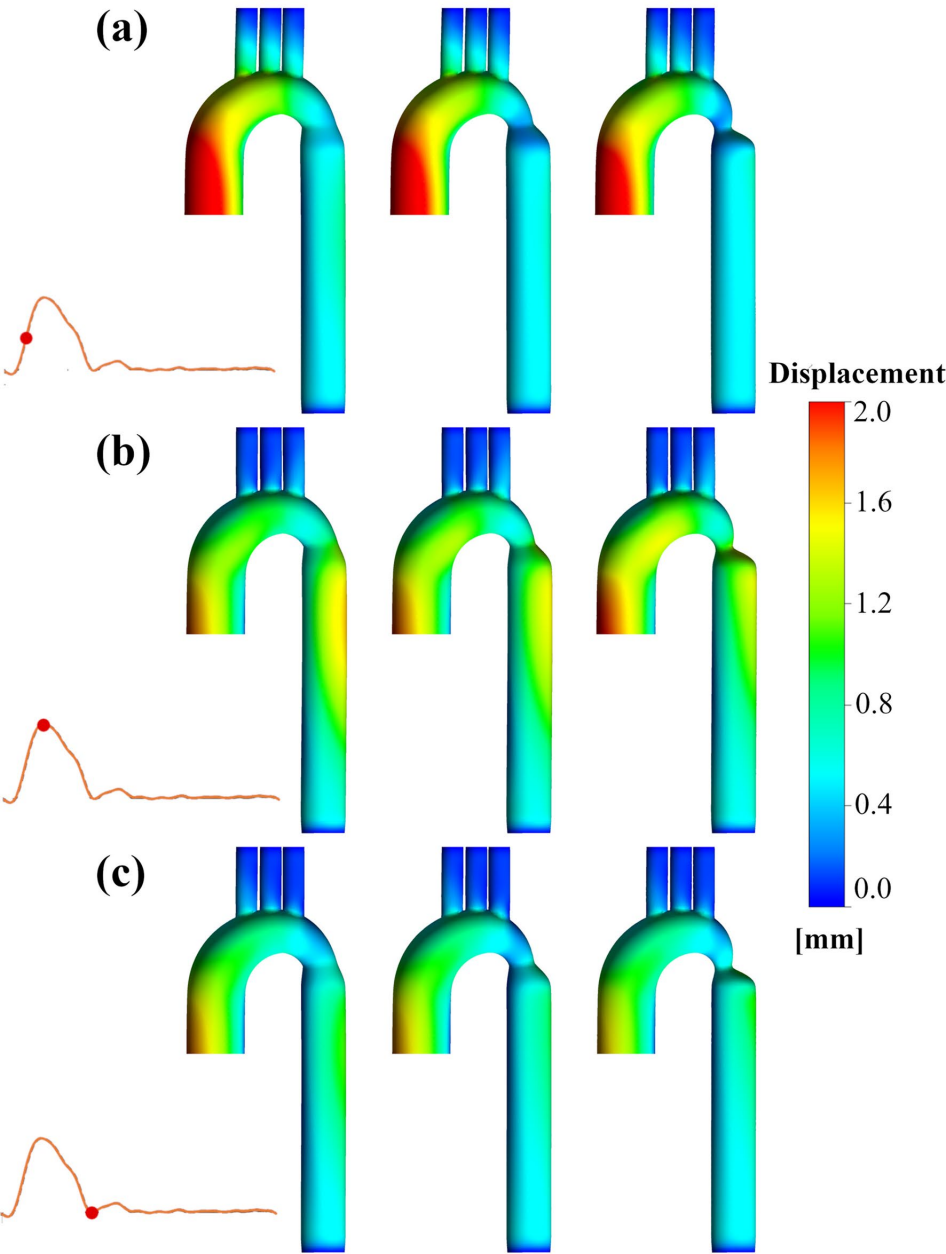
Vessel wall displacement during the cardiac cycle. Contours demonstrate the displacement of the geometry in the right anterior view at (**a**) mid-systole, (**b**) peak systole, and (**c**) dicrotic notch (left: 25% coarctation, middle: 50% coarctation, right: 75% coarctation).

The aortic wall’s relocation for three coarctation cases is depicted in Fig. 2 at three instances of time. Figure 2a indicates the displacement (the translational motion) during mid-systole for three coarctation degrees (left: 25% coarctation, middle: 50% coarctation, right: 75% coarctation). The ascending aorta has been displaced outwards, that its volume has increased in response to increased blood flow rate. The biggest deformation of the entry can be seen in the mild coarctation, by up to 3.07 mm. This indicates that the narrowed region acts as a barrier to the deformation of the ascending aorta during flow entrance. At peak systole, also three right anterior views of three cases are shown (Fig. 2b). A more noticeable displacement at the entry region is observed for the severe case in comparison to the milder ones.

### Velocity distribution

**Figure 3:**
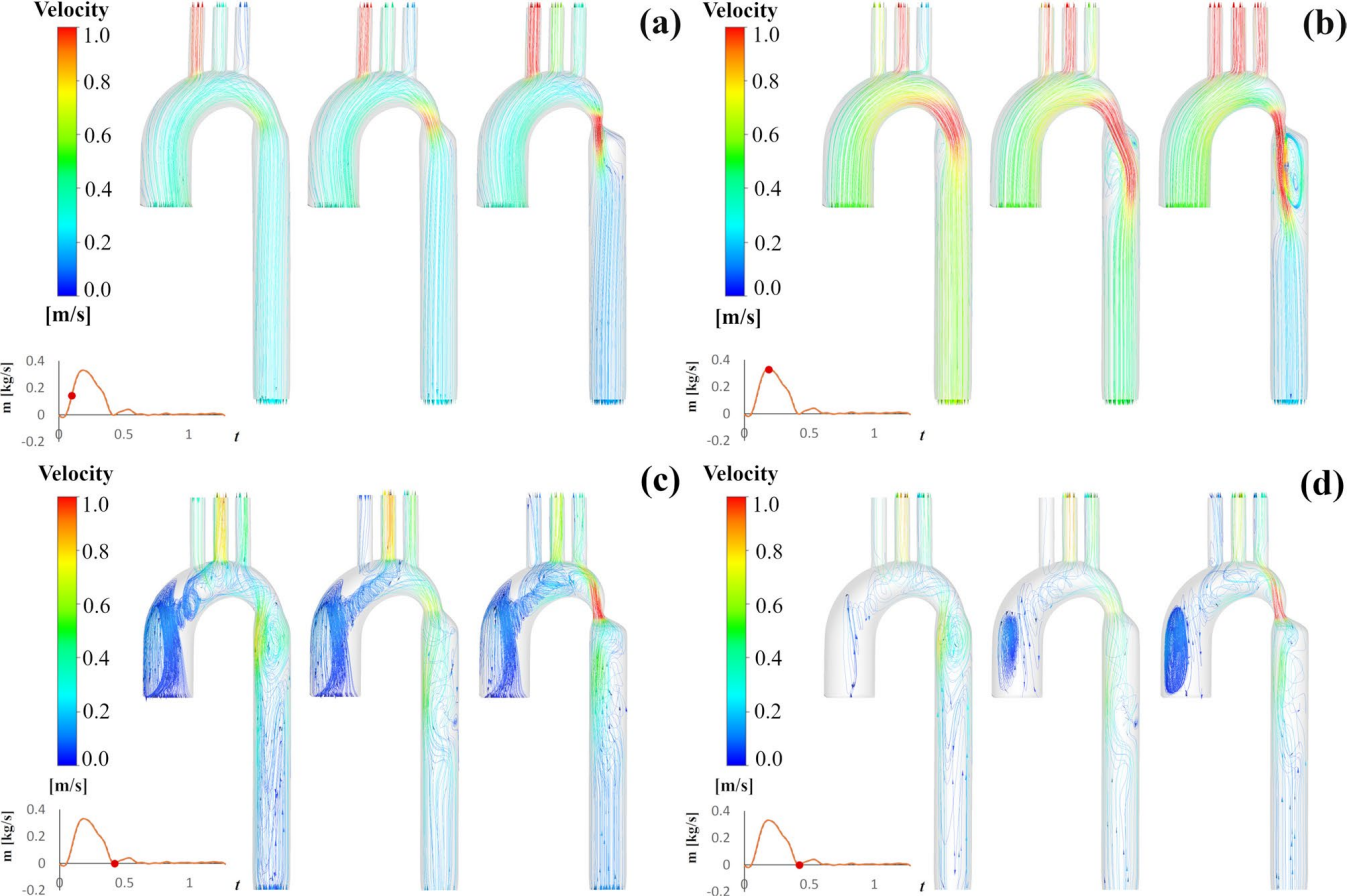
Streamlines in the right anterior view at (**a**) mid-systole, (**b**) peak systole, and (**c**) dicrotic notch; (**d**) Backward flow during dicrotic notch (left: 25% coarctation, middle: 50% coarctation, right: 75% coarctation).

Figure 3 indicates the forward and backward streamline patterns that the magnitude of the velocity of the flow is shown in color during the cardiac cycle for three different CoA degrees. During mid systole (Fig. 3a), uniform streamlines are demonstrated along the branches, aortic arch, and the CoA zone. After the coarctation, highly in the most severe case, the streamlines become less regular by a loss of symmetry, and some vortices are observed. During peak systole (Fig. 3b), in the moderate case, low-velocity irregular streamlines are seen after the CoA area. As the coarctation severity increases, it is clearly shown that streamlines become more irregular and more vortices have been generated, and velocity magnitude at the narrowing area increases. The separation zone right after the tapered part exists in all three cases. However, it’s negligible for mild one and becomes more as the coarctation degree increases. As the narrowing degree increases, the high-velocity jet striking the descending aorta wall becomes more considerable, and also, high-velocity streamlines affect the branches more. During the dicrotic notch, disordered streamlines across the ascending aorta and vortices in the entry and arch and after narrowing can be seen. The considerable-scale helical flow structures have been developed in the ascending aorta (Fig. 3c), and also a jet formation at CoA can be seen in the severe case. Figure 3d shows the backward flow for three CoA cases during dicrotic notch. Stiffening and a local narrowing causes wave reflections quickly reaching the heart^5^, which can be clearly seen in this study.

**Figure 4:**
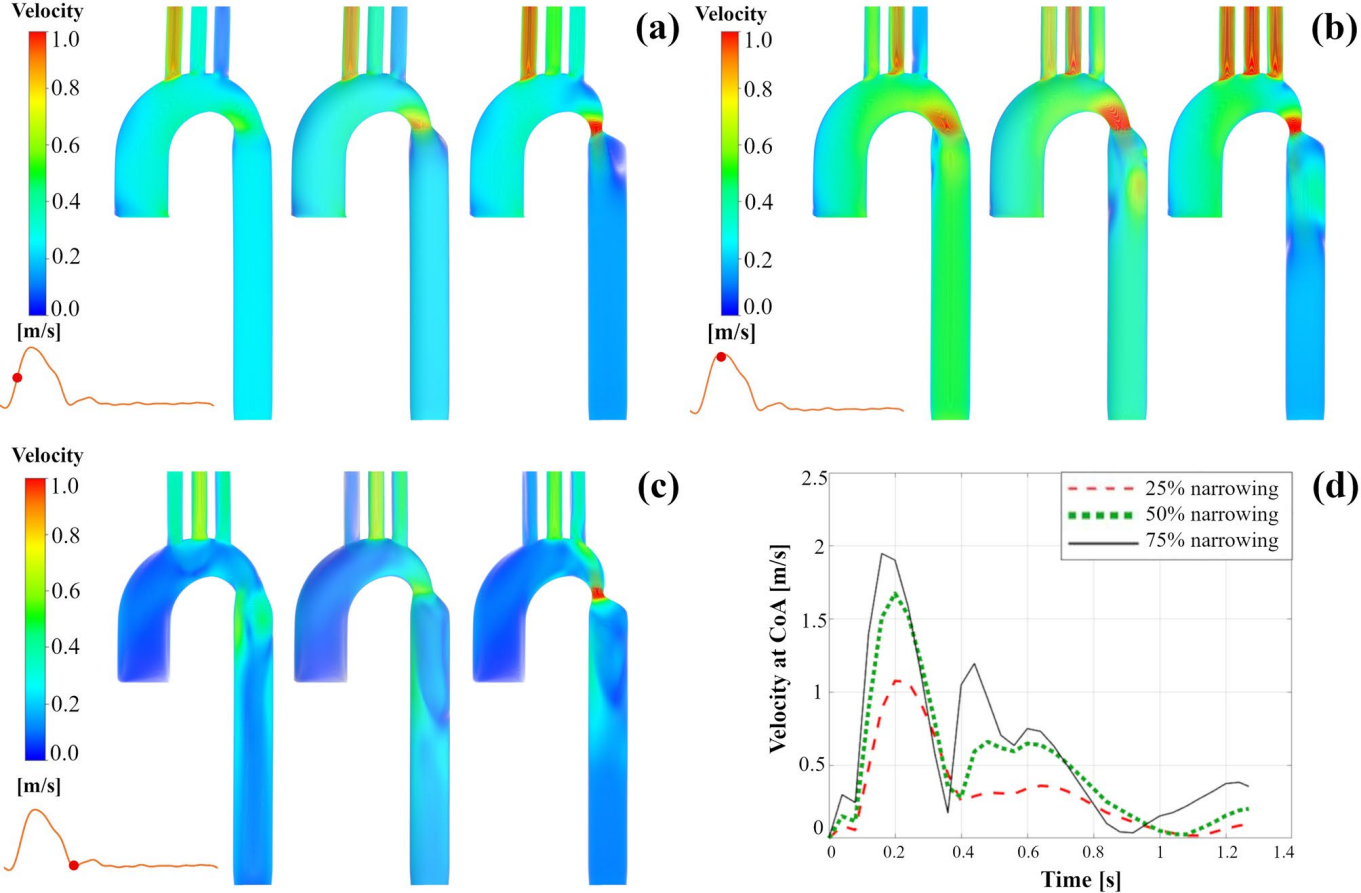
Velocity volume renders at various time instances. Figure shows the velocity magnitude in the right anterior view at (**a**) mid-systole, (**b**) peak systole, and (**c**) dicrotic notch (left: 25% coarctation, middle: 50% coarctation, right: 75% coarctation); (**d**) Velocity at CoA in three cases against time.

The velocity profile for three coarctation cases at three time instances are shown in Fig. 4. Blood acceleration across the coarctation region, specifically in moderate and severe cases, produced a downstream swirling, with a high-velocity jet striking the descending aorta wall. The maximal velocity increases up to 1.07 m/s, 1.67 m/s, and 1.95 m/s (shown in Fig. 4d), respectively for three cases, as the CoA area becomes narrower. High blood velocities are observed in the narrowing section, where the coarctation causes a reduction in the cross-sectional area. Furthermore, each supra-aortic vessel displayed evidence of increasing velocity magnitude as the coarctation became more severe.

### Pressure distribution

**Figure 5:**
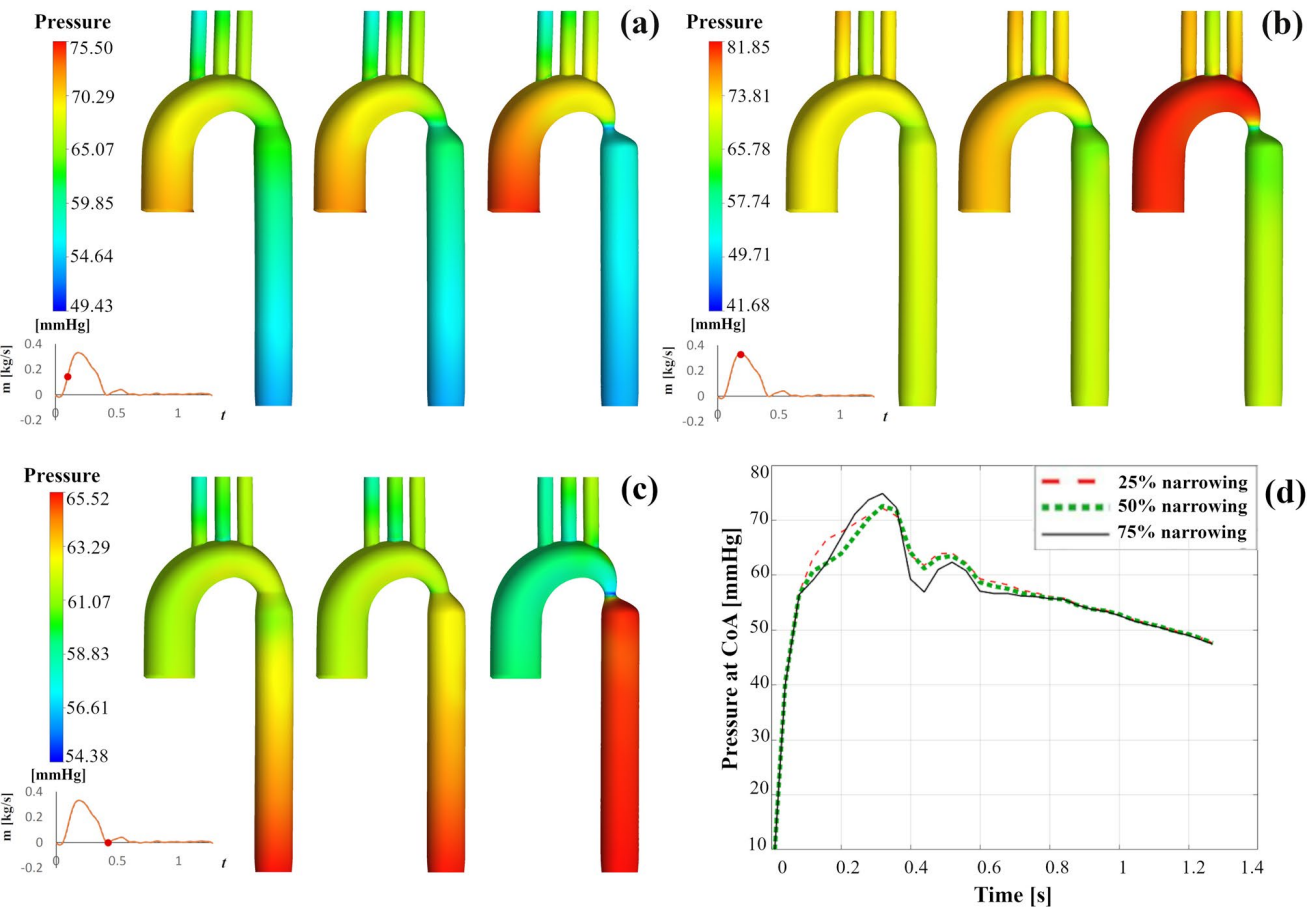
The distribution of pressure in the right anterior view at (**a**) mid systole, (**b**) peak systole, and (**c**) dicrotic notch (left: 25% coarctation, middle: 50% coarctation, right: 75% coarctation), (**d**) pressure distribution in CoA area in three cases against time.

The pressure distribution along the aorta is depicted during the heart cycle in Fig. 5. Figure 5a illustrates the distribution of pressure during mid-systole for three coarctation cases. Along with the ascending aorta, the pressure is highest, then drops at the CoA and the supra-aortic branches. As the coarctation severity increases, the difference between ascending and descending aorta's pressure becomes larger. Figure 5b indicates the distribution of the pressure at the maximum systole, which is similar to that of mid-systole. However, the gradients of intraluminal pressure are more pronounced. During the dicrotic notch (Fig. 5c), the aortic arch is linked to the lower pressure, whereas the descending aorta is linked to the higher pressure. Figure 5d represents the pressure at the coarctation area against time for three cases. Figure 5 also shows that pressure falls sharply due to the narrowing. Along the CoA’s distal end, the deceleration of flow is followed by pressure retrieval over the entire descending aorta.

### Wall shear stress and oscillatory shear index

**Figure 6:**
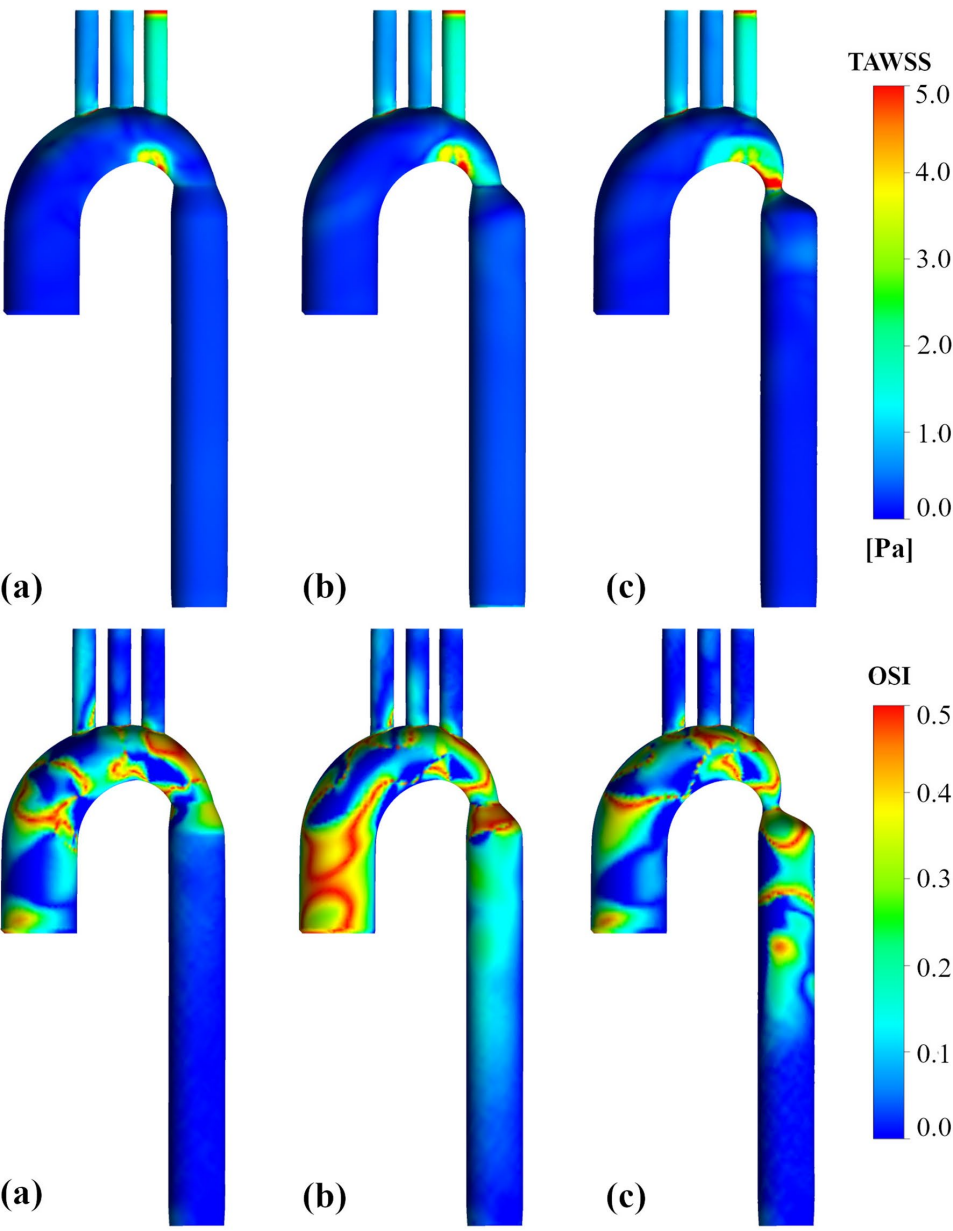
TAWSS and OSI characteristics for the FSI simulation. (**a**) 25% coarctation (**b**) 50% coarctation (**c**) 75% coarctation.

WSS prediction is one of the key outputs from FSI simulations, usually analysed by applying indices such as TAWSS and OSI. The distribution of TAWSS, obtained with FSI simulations for three coarctation cases during the cardiac cycle, is shown in Fig. 6. As much as the coarctation becomes more severe, high TAWSS magnitudes affect the narrow part more, and the difference between TAWSS magnitude before and after coarctation becomes more prominent.

While OSI represents the WSS's changes during a cardiac cycle, it does not account for the WSS magnitude; thus, it is utilized with TAWSS. High OSI values exist throughout the aortic arch up to the CoA zone in all three cases, which means that the changes of direction are larger than the mean flow there. High OSI values also exist exactly after the narrowing part. However, the more severe the coarctation becomes, the more part after the coarctation is affected; high OSI has affected considerably more parts of the descending aorta for the severe CoA. What is more, some helical areas of high OSI are generally seen in areas with low TAWSS. Flow recirculation in these areas contributed to low TAWSS and high OSI.

### HOLMES

**Figure 7:**
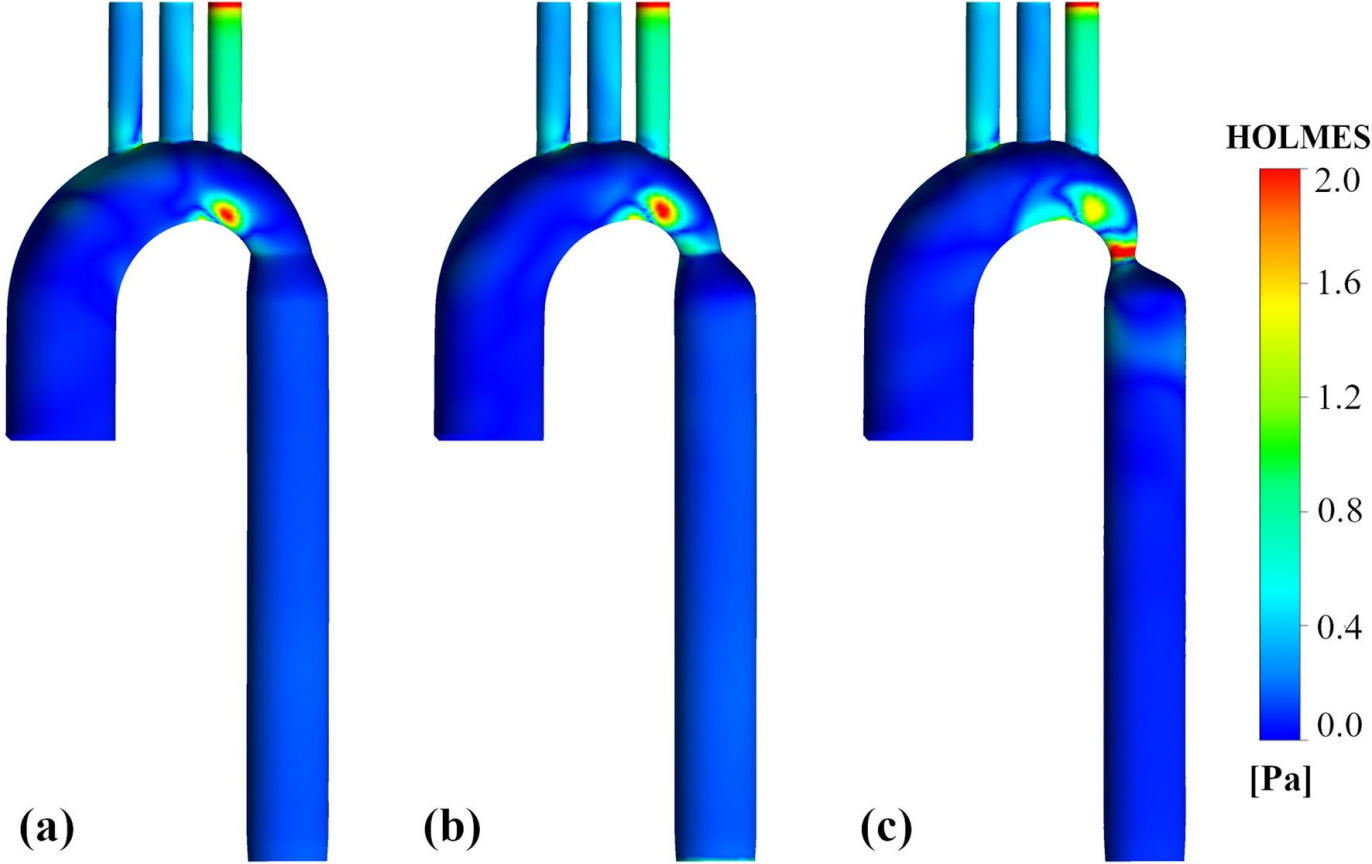
HOLMES characteristics. HOLMES distributions for the FSI simulation. (**a**) 25% coarctation (**b**) 50% coarctation (**c**) 75% coarctation.

Figure 7 depicts the distribution of the HOLMES index for three cases, which has a strong correlation with TAWSS distribution. The HOLMES magnitude varies before the coarctation zone, and it suddenly changes after the CoA, especially in the most severe case. Maximum amounts of HOLMES become closer to the narrowing as the CoA becomes more severe. According to Fig. 6, some regions show high TAWSS and low OSI that have been recognized as high-risk areas for the rupture of an aneurysm^53, 54^, which are shown in Fig. 7 as areas with high amounts of HOLMES.

### RRT

**Figure 8:**
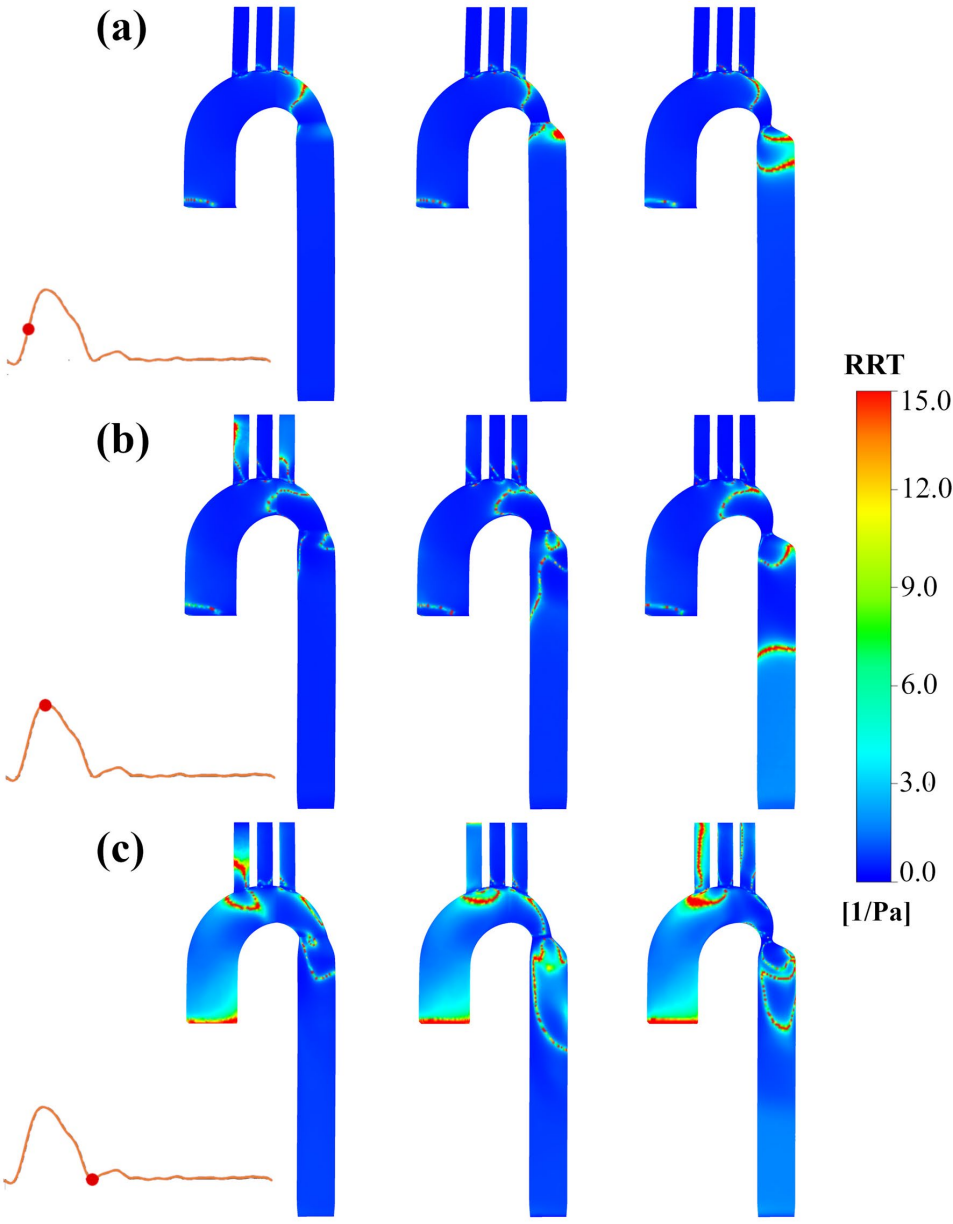
Contours of relative residence time (RRT) characteristics. RRT distributions for the FSI simulation (**a**) at mid-systole, (**b**) at peak systole and (**c**) at dicrotic notch (left: 25% coarctation, middle: 50% coarctation, right: 75% coarctation).

The contours of RRT under the three-time instances for three coarctation degrees are shown in Fig. 8. RRT is increased right after the CoA and helical areas before the CoA near the left subclavian artery at mid-systole (Fig. 8a), in the middle of the aortic arch at peak systole (Fig. 8b), and the concave part of the arch downstream to brachiocephalic artery at the dicrotic notch while branches were also affected (Fig. 8c). The more severe the coarctation gets, high RRT affects the farther distances from the CoA along the descending aorta. The RRT distribution at mid-systole and peak systole were almost the same. However, some additional parts, including branches and the area near the brachiocephalic artery entry, have large RRT values at the dicrotic notch.

## Discussion

Despite a general thought of CoA as a simple disease, evidence shows a reduced lifespan compared to the normal population^55^. Therefore, the assessment of this disease for the purpose of treatment is essential. CoA's complex flow quantification plays a critical role in precise diagnosis, assisting the physicians in optimizing the interventions and better risk stratification. To expand anatomical information, clinicians rely mainly on cardiac catheterization data to assess flow and pressure through the circulatory system, instead of sophisticated methods such as fluid dynamics, but this is invasive, high risk, and expensive^56, 57^. MRI may provide a 3-D velocity field, but it is costly and impossible for many patients with a low temporal resolution^30, 58–60^.

In this study, three different CoA degrees have been developed, and an FSI model is applied to evaluate the CoA severity's impact on haemodynamics correctly. This is while most CoA studies are conducted under the assumption of the rigid wall, and this oversimplification can distort insights^17–25^. Simulations with rigid walls fail to capture some crucial physiological patterns (such as wave propagation and reflection), which lead to significantly different predictions of WSS indices. Studies^41^, have shown a considerable difference in WSS indices (by more than 50%) in the absence of moving walls. Thus, the inclusion of walls is invaluable in such studies.

Arterial distensibility is an essential factor for assessing cardiovascular disorders, as raised vascular stiffness is associated with an increased risk of cardiovascular disease^6–9, 61^. The smaller cross-section of the CoA zone results in higher blood velocity, contributing a more focused flow jet in the severe case that affects the aortic wall and causes dilatation of the ascending aorta^61^. The maximum displacement, located in the ascending aorta due to the heart’s blood pump, is greater for the severe CoA case, which was also seen in other studies^30^.

It can be seen that the narrower the CoA area, the wave reflections reach the heart more quickly. Having said that, CoA leads to a significant impedance discrepancy between the two sections of the stenotic thoracic aorta and the final aortic arch, with an increased reflection of wave^17^ and quick return of the reflected pressure wave, which increases proximal pressure. Khir and Parker have reported similar findings regarding the aortic obstruction in dogs^62^.

As the coarctation severity progresses, the pressure difference between ascending and descending aorta increases. Earlier results of a study in dogs with synthetic CoA also confirmed that the aortic pressure during peak systolic increased considerably with the aorta's progressive coarctation^63^.

An intense decrease in flow through the thoracic aorta can also be seen, which tends to a more irregular profile. This leads to the generation of downstream swirling, with a high-velocity jet hitting the descending aorta wall, contributing to high and unidirectional WSS vectors within the narrowing area. Findings show the indispensability of recognizing the coarctation degree due to the surprising TAWSS values differences in the CoA area. TAWSS increases considerably with the CoA progression, and the most apparent differences can be seen in close to zero OSI areas corresponding to high TAWSS values, located before the coarctation area. Also, others have reported the OSI’s inability to capture disturbances in areas of elevated TAWSS, which eventually cause rupture^53, 54, 64^.

As low TAWSS and raised OSI are associated with those likely to atherosclerotic plaque development^65–67^, it can be realized that two particular areas are in danger of plaques creation: before the coarctation area with a specific distance, which does not change with the change of the CoA degree, and immediately after the narrowing, which expands as the coarctation becomes more severe. In regard to the first area, The helical regions in which TAWSS magnitude suddenly decreases and OSI increases are at the aortic arch, associated with high RRT, which is seen in other studies too^64, 68–70^. This favors cell–cell interactions and strengthens cell adhesion probability on vessel walls^71^. Regarding the latter area, the flow separates from the aortic wall exactly after leaving the tapered region. It goes through vortex formation leading to low-velocity flow, which eventually provoked low and multidirectional WSS vectors, leading to high RRT at the descending aorta, seen in other studies too^72–74^. For the severe CoA case, RRT contours show the disturbed hemodynamics in the lower parts of the descending aorta, which means that a larger portion of the vessel needs to be treated.

A combined index named HOLMES, besides the regular TAWSS or OSI, is applied to indicate highly oscillatory, low WSS areas, in agreement with many findings suggesting these locations as especially harmful for the endothelium^55^. The degree of narrowing caused HOLMES reduction precisely after the CoA zone significantly (up to 75%), which is more considerable for the severe case in comparison to the milder ones.

This provides a platform for improving CoA therapy on a patient-specific level, in which physicians can perform treatment methods based on WSS indices.

To recapitulate, reaching a routine based on WSS indices on top of experience for optimized treatment for coarctation patients is the main purpose of this study. This started by comparing the different degrees of coarctation. Results show how severe CoA affects the aorta in comparison to the milder cases that indicate the fact that this fluid–solid interaction simulation approach can give the medical community valuable information. Therefore, this information can develop our knowledge of this disease and, by applying it to each patient, can help us make better decisions. As future progress, the framework based on WSS indices would be benchmarked with many clinical cases before and after treatment. Results of the present study demonstrate the ability of the framework to track changes in aortic disease status before and after any intervention. The present study is the first FSI study that emphasizes the usage of WSS indices for CoA patients.

### Limitations

The model presented here assumes a homogeneous elasticity at the wall. As future progress, further FSI studies are required to assess different severities on patient-specific cases. The framework based on WSS indices would be benchmarked with many clinical cases before and after treatment; this can raise the idea for using WSS indices for clinical usage and predictions and repair planning.

## Data Availability

All data used for this study are available from the author upon request.
